# Stratification of surgical outcomes in trigeminal neuralgia using multimodal data

**DOI:** 10.1093/braincomms/fcaf178

**Published:** 2025-06-17

**Authors:** Timur H Latypov, Rose Yakubov, Daniel Jörgens, Pascale Tsai, Patcharaporn Srisaikaew, Peter Shih-Ping Hung, Matthew R Walker, Marina Tawfik, David Mikulis, Frank Rudzicz, Mojgan Hodaie

**Affiliations:** Krembil Research Institute, University Health Network, Toronto, Ontario, Canada, M5T 0S8; Institute of Medical Science, Temerty Faculty of Medicine, University of Toronto, Toronto, Ontario, Canada, M5S 3K3; Collaborative Program in Neuroscience, University of Toronto, Toronto, Ontario, Canada, M5S 3K3; MD Program, Temerty Faculty of Medicine, University of Toronto, Toronto, Ontario, Canada, M5S 3K3; Krembil Research Institute, University Health Network, Toronto, Ontario, Canada, M5T 0S8; Michael G. DeGroote School of Medicine, McMaster University, Hamilton, Ontario, Canada, L8P 1H6; Krembil Research Institute, University Health Network, Toronto, Ontario, Canada, M5T 0S8; Krembil Research Institute, University Health Network, Toronto, Ontario, Canada, M5T 0S8; Institute of Medical Science, Temerty Faculty of Medicine, University of Toronto, Toronto, Ontario, Canada, M5S 3K3; Collaborative Program in Neuroscience, University of Toronto, Toronto, Ontario, Canada, M5S 3K3; Krembil Research Institute, University Health Network, Toronto, Ontario, Canada, M5T 0S8; Collaborative Program in Neuroscience, University of Toronto, Toronto, Ontario, Canada, M5S 3K3; Department of Computer Science, University of Toronto, Toronto, Ontario, Canada, M5S 2E4; Vector Institute for Artificial Intelligence, Toronto, Ontario, Canada, M5G 0C6; Krembil Research Institute, University Health Network, Toronto, Ontario, Canada, M5T 0S8; Joint Department of Medical Imaging, Toronto Western Hospital, University Health Network, Toronto, Ontario, Canada, M7A 2S4; Department of Computer Science, University of Toronto, Toronto, Ontario, Canada, M5S 2E4; Vector Institute for Artificial Intelligence, Toronto, Ontario, Canada, M5G 0C6; Faculty of Computer Science, Dalhousie University, Halifax, Nova Scotia, Canada, B3H 1W5; Krembil Research Institute, University Health Network, Toronto, Ontario, Canada, M5T 0S8; Institute of Medical Science, Temerty Faculty of Medicine, University of Toronto, Toronto, Ontario, Canada, M5S 3K3; Department of Neurosurgery, Toronto Western Hospital, University Health Network, Toronto, Ontario, Canada, M5T 2S8

**Keywords:** machine learning, chronic pain, neuroimaging, trigeminal neuralgia, diagnosis and prognosis

## Abstract

Chronic pain remains a challenge for clinicians, with limited individualized predictive tools that can aid with diagnosis, disease course, or prediction of treatment outcomes. We hypothesized that a comprehensive analysis, encompassing a patient's complete pain-related clinical data, medical history and brain imaging, can identify key contributors linked to surgical outcomes and stratify specific outcome categories for trigeminal neuralgia (TN)—chronic facial pain syndrome. Using supervised and unsupervised machine learning approaches, we analysed data from 102 subjects with classical TN. Pre-surgical clinical data were processed through unsupervised learning to delineate key clinical contributors of TN outcome stratification and their correlation with surgical response. Concurrently, we applied supervised learning to pre-surgical T1-weighted brain magnetic resonance imaging. Clinical data analysis uncovered pain and non-pain-related measures—including pain frequency, degree of medication relief, pain character, presence of diabetes and cancer history—as the most significant in forecasting surgical outcome. Analysis revealed strong correlation of pre-surgical clinical data with surgical response duration (*r* = 0.5, *P* < 0.00001). Imaging data analysis used a support vector machine classification model with high recall for subjects who would be either long-term responders or non-responders 0.79 and 0.86 with the area under the receiver operating characteristic curve (AUC) of 0.86 and 0.84, respectively. The average multiclass accuracy in predicting the duration of surgical response categories was 78% (AUC 0.8). Together, these results show that TN surgical outcome categories are distinguishable, and surgical outcome can be stratified based on combined clinical and brain imaging data available prior to surgical treatment. We suggest a novel perspective on different strata of chronic pain disorders, each with structural imaging, clinical correlates and specific surgical outcomes.

## Teaser

Latypov *et al*. report that machine learning models analysing clinical and brain imaging data predict duration of surgical outcomes for chronic facial pain. Their findings offer novel insights into personalized pain assessment, demonstrating that surgical success can be anticipated pre-operatively using patient-specific multimodal data.

## Graphical Abstract

**Graphical Abstract:**
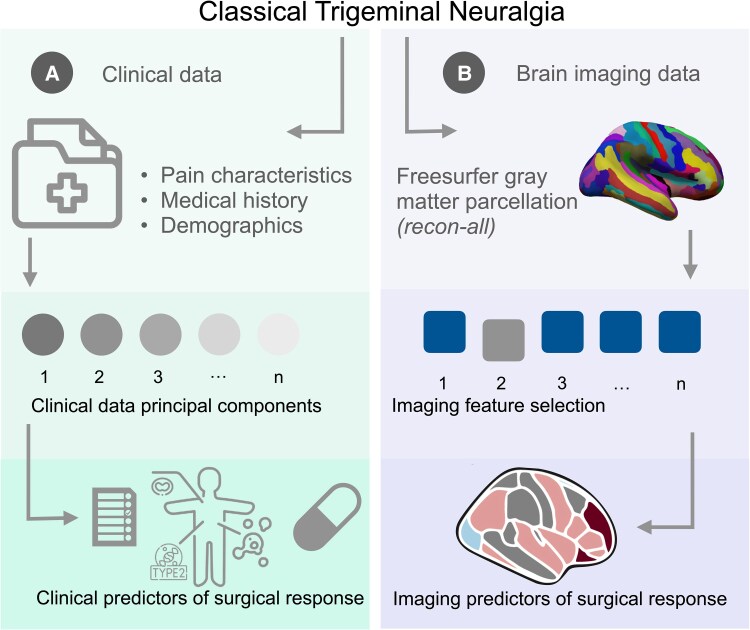

## Introduction

Trigeminal neuralgia (TN) is a chronic unilateral facial pain condition associated with one or more divisions of the trigeminal nerve (CN V) distribution. Patients with classical TN experience paroxysmal pain that is described as electric or shock-like in nature. A proportion of patients also experience longer bouts of pain, or a background of milder, constant pain, which is described as burning or dull. On this basis, the international classification of headache disorders 3rd edition (ICHD-3) subdivides classical TN into purely episodic TN subtype and TN with concomitant pain.^1^ The variation in the expression of pain, however, goes beyond outlined subtypes,^2,3^ suggesting the need to stratify TN into disease categories that may account for the clinical features of TN and inform surgical outcomes.

Patients who are refractory to medication treatment^4^ are typically considered for surgery such as Gamma Knife radiosurgery (GK), microvascular decompression (MVD), or percutaneous rhizotomy. Although surgical treatment can provide prolonged pain relief in >70% of patients, a proportion of patients still experience inadequate pain relief following surgery.^5,6^ Stratification of surgical outcomes based on presenting symptoms has been suggested^2,7^ but has been of limited practical value, given the number of variables (clinical and demographic) that may factor in the definition of the strata.

Machine learning (ML) has the capacity to address this technical limitation, as these approaches are able to perform assessment based on TN clinical characteristics and brain imaging signatures, therefore facilitating such stratification.^8-10^ ML has been previously employed to investigate advanced brain imaging signatures of TN.^11-13^ The utilization of clinical data using ML to study TN has not been done before. This is especially important, since there is a lack of individualized predictors and prognosticators of treatment response for chronic pain patients that could rely on both patient's clinical data and objective imaging signatures.^14^ Relating these measures to the surgical outcome of TN can bridge this gap, paving the way towards stratifying categories of surgical outcome in TN and identifying measures (both clinical and imaging-based) that are predictive and prognostic for the surgical outcome.

We hypothesize that TN can be defined as a pain syndrome that includes a spectrum of outcome categories determined by the combination of individual pain characteristics, clinical history and brain imaging signatures. In this study, we define each category based on different surgical outcomes (i.e. distinct categories of surgical response duration) and analyse clinical and imaging data to stratify these categories of TN surgical response. We address the hypothesis with a multimodal study, where we propose a stratification of categories of classical TN based on pre-surgical brain imaging and clinical data and identifying clinical and imaging contributors for each stratum.

## Materials and methods

### Patient recruitment

This retrospective study was approved by the University Health Network Research ethics board. A total of 102 patients, aged between 24 and 85 and initially diagnosed with TN, defined by the ICHD-3 were included in this study (ICHD-3 ID 13.1.1).^1^ All patients had a clinical and imaging follow-up (f/u) before receiving surgical treatment for TN between 2005 and 2018. We included patients who underwent GK or MVD surgery. Intervention was chosen independently by the neurosurgical team strictly based on clinical guidelines and surgical indications, including presence of the neurovascular conflict, presence of any contraindications for craniotomy or severe comorbidities.

Inclusion criteria were age between 18 and 85, presence of the unilateral pain, duration of TN pain for more than 6 months. Patients with TN that were secondary to multiple sclerosis, brain tumour, traumatic nerve injury, or infection were excluded from this study. We also excluded patients with TN who previously had percutaneous rhizotomy for TN pain as well as patients with bilateral TN, solitary pontine lesion TN^15^ and a history of neurological disorders (multiple sclerosis, Alzheimer's disease, amyotrophic lateral sclerosis). We included patients who were actively taking medication for TN, including carbamazepine, oxcarbazepine, gabapentin and pregabalin.

A successful surgical response (Rs) was defined as ≥75% reduction in pain intensity, assessed by the 11-point pain numeric rating scale, and a score of I–III on the Barrow Neurological Institute (BNI) scale. As the onset of pain relief is different for GK and MVD surgeries, the initial response was evaluated 3 months after surgery for patients who underwent MVD and 6 months for ones who were treated with GK. Non-responders (NR) were defined at these time points, while responders were followed up until pain reoccurrence (self-reported return of BNI IV–V pain) or for 5 years from the date of surgery. Patients who experienced pain recurrence within 1 year since the surgery date were identified as short-term (StR) responders (<1-year responders). Mid-term responders (MtR) had pain relief for 1–3 years after surgery. Long-term responders (LtR) included patients with more than 3 years of pain relief following surgery. Within this LtR group, we defined two subgroups: (i) patients that experienced 3–5 years of pain relief; and (ii) patients who reported complete pain relief with no evidence of recurrence for >5 years after surgery.

### Data collection

#### Magnetic resonance image acquisition and processing

Pre-surgical treatment anatomical T1-weighted images (fast-spoiled gradient echo, TE = 5.1 ms, TR = 12.0 ms, flip angle = 20°, voxel size = 0.86 mm × 0.86 mm × 1.00 mm, 256 × 256 matrix, field of view = 22 cm, 146 slices) were acquired for each patient. We used a 3 tesla GE Signa HDx magnetic resonance imaging scanner (General Electric Healthcare, Milwaukee) with an eight-channel head coil. We used FreeSurfer (v7.2) automatic parcellation software to process all images and extract a set of regional cortical and subcortical grey matter (GM) metrics.^16^ These include cortical surface area, cortical thickness^17^ and subcortical GM volumes such as thalamic nuclei,^18^ hippocampus and amygdala,^19,20^ totalling 445 GM metrics. Following extraction, all metrics were corrected for individual differences in head size using estimated total intracranial volume.

#### Clinical data for feature extraction

Pre-surgical clinical variables, including pain characteristics, demographic features and medical history information, was collected for each patient from the University Health Network electronic patient records. Pain characteristics of interest included side, duration, description of pain, triggers, attack frequency, CN V division(s) affected, subjectively reported numbness and pain medication effect (eight variables). Medical history was evaluated by neurosurgical history and comorbidities at the time of pre-surgical f/u grouped by the affected organ system (15 variables). Such clinical variables are routinely collected at pre-operative consultations for TN patients to support clinician decision-making and treatment planning. With age and sex as demographic variables, a total of 25 clinical variables were collected for the analysis. Self-reported rank variables were one-hot encoded, including frequency of attacks and self-reported degree of medication relief. A total of 36 clinical and demographic features from 25 variables were extracted for the analysis (Supplementary Table 1).

### Machine learning and statistical analyses

**Figure 1:**
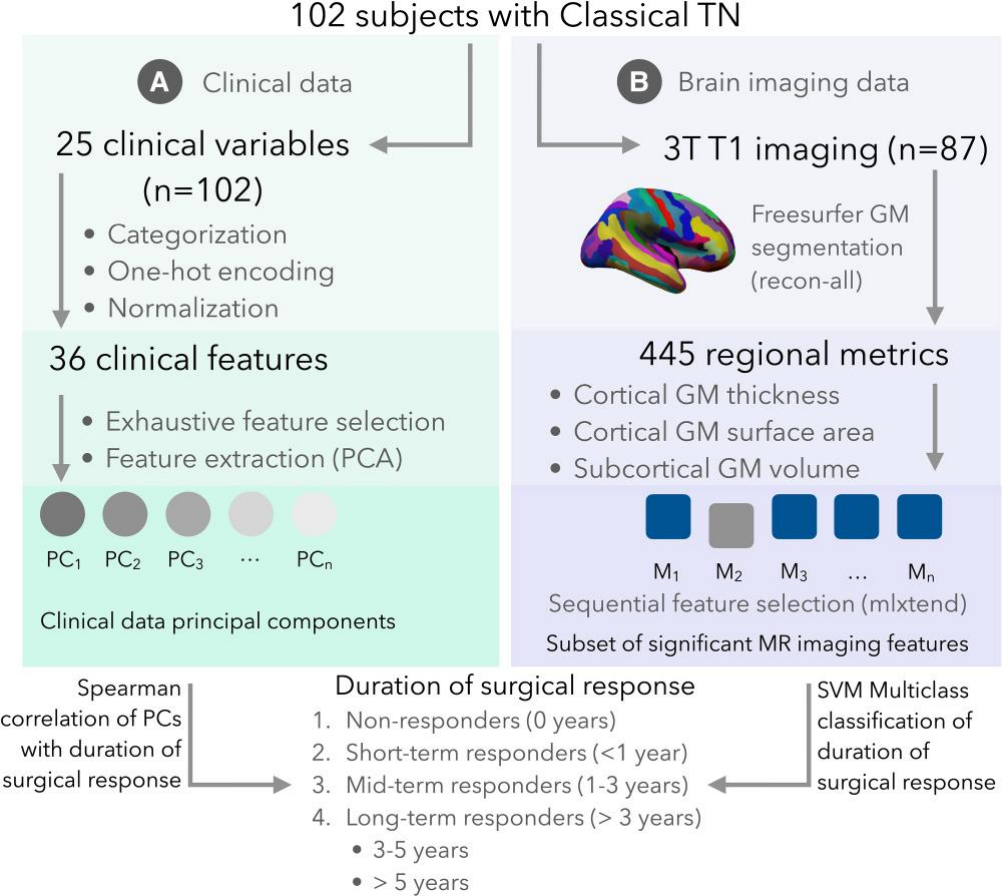
**Study design summary.** (**A**) Clinical data branch—clinical data underwent transformation to categorical features, one-hot encoding and normalization. PCA and exhaustive feature selection were conducted. The set of features that demonstrated the strongest correlation with surgical response duration was determined. (**B**) Imaging branch—T1 data were processed in FreeSurfer, and regional grey matter metrics were extracted. Sequential feature selection and the SVM classification model were used to predict the duration of surgical response. GM, grey matter; MR imaging, magnetic resonance imaging; PCA, principal component analysis; SVM, support vector machine; TN, trigeminal neuralgia.

This study uses two different approaches to identify predictors of duration of surgical response. Unsupervised ML using principal component analysis (PCA) was used with Spearman correlation to identify the clinical prognosticators with the highest impact on the category of surgical response (Fig. 1A, Supplementary Content P1). Supervised ML approach uses grey matter imaging metrics and multiclass support vector machine (SVM) classifier model to predict the category of surgical response (Fig. 1B, Supplementary Content P2). Feature selection methods were applied to both analyses to identify the optimal combination of features. We followed sequential feature selection approach that was previously used to distinguish responders and non-responders to the GK surgery in TN.^12^ In order to confirm generalizability, we used nested cross-validation (CV) approach. Outer CV was used for out-of-sample evaluation, and inner CV was utilized for feature selection and hyperparameter tuning.

Supervised and unsupervised learning branches were separated, and we did not attempt to combine data. This has been done in order to ensure that one type of data is not introducing bias to the model.^21,22^ We do not aim to determine if one type of data is giving a better prediction over the other. Instead of combining categorical clinical data and brain imaging or compare these two modalities, we used the common outcome variable (duration of response to TN surgery) to independently assess clinical and imaging prognosticators of surgical outcome in corresponding analyses.

Univariate *post hoc* statistical tests were performed to identify the directionality of neuroimaging predictors (Supplementary Content P3). We used GM features that were consistently (at least 6/10 times) identified as important across 10 folds. These GM metrics were compared between TN subjects and age- and sex-matched healthy controls (HC) from Cambridge Center for Ageing Neuroscience dataset (*n* = 87).^23^ Previous studies confirmed that FreeSurfer GM metrics are less susceptible for the cross-scanner difference.^13,24^ As such, we did not apply harmonization methods to GM data.

## Results

### Patient demographics

The study population includes 43 males and 59 females, 37 left-sided TN (L-TN) and 65 right-sided TN (R-TN). Of these, 49 patients reported pain relief and recurrence of symptoms within 5 years after surgery, 20 patients remained pain-free for more than 5 years after the surgery, and 33 were categorized as non-responders TN (NRs). Fifteen subjects were excluded from the supervised learning analysis due to processing errors and imaging artefacts, therefore, a totalling of 87 subjects were used in supervised learning on MRI data. No statistically significant difference was found in the proportion of surgical response between the GK and MVD groups (two-proportion z-test stat = −2.07, *P* > 0.05). A summary of demographic information is outlined in Table 1.

**Table 1:** Demographic information of the study population

Dataset demographics	Initial diagnosis of TN (ICHD-3 code 13.1.1)
Sample size (n)	102
Age (years, y)	60.3 ± 14.4
Sex (males:females)	43:59
Pain side (L:R)	37:65
Surgery (GK:MVD)	70:32
Surgery outcome	
Non-responders (n)	33
Recurrence within 5 years after surgery (n)	49
Pain-free for more than 5 years (n)	20

### Clinical signatures of post-surgical pain relief prognosis

**Figure 2:**
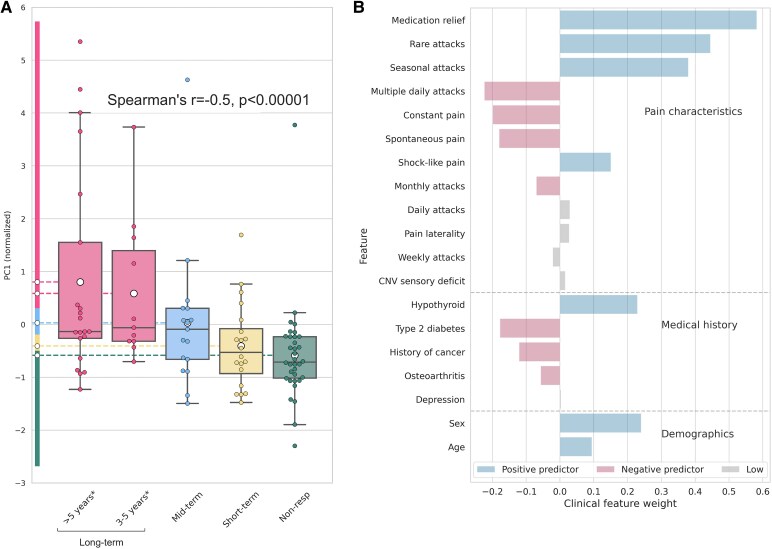
**Relationship between clinical variables and surgical outcome in TN.** A combination of 19 normalized clinical features is strongly and significantly correlated with the duration of TN surgical response in a principal component (PC1) that is strongly and significantly correlated with the duration of TN surgical response classes (Spearman rho = 0.5, *P*-corrected < 0.0001, *n* = 102). (**A**) PC1 and different durations of surgical response (non-responders, short-term, mid-term and long-term); each data point represents PC1 value for individual subject (score composed of 19 clinical features); (**B**) PC1 feature weights derived from principal component analysis (PCA) based on data from 102 patients (*n* = 102). Features are ranked according to their loadings on the first principal component (PC1; PC1 eigenvalue = 2.11). Higher absolute values of weights indicate a greater contribution of the clinical feature to PC1. Positive predictors are associated with higher PC1 scores and longer durations of surgical response, whereas negative predictors linked to lower PC1 scores and shorter response durations. Features with low absolute weight values (<0.05) represent minimal contribution. Non-resp, non-responders TN; PCA, principal component analysis; PC1, principal component 1; TN, trigeminal neuralgia.

Transformed pre-surgical clinical features (36 features, *n* = 102) underwent exhaustive feature selection, followed by PCA transformation. Exhaustive feature selection identified a set of 19 important clinical and demographic features. Seventeen PCs were extracted from the set and PC1 showed the most significant and strongest correlation with the duration of surgical response (Spearman's *r* = 0.48, corrected *P* < 0.0001; see Fig. 2A). Subgroups of LtR with 3–5 years of pain relief and >5 years of pain relief showed high similarity in the PCA correlation analysis as demonstrated by the overlapping distributions (see Fig. 2). Combining these two groups in one improved strength of correlation (Spearman's *r* = 0.5, corrected *P* < 0.0001). For that reason, we considered LtR a single group of >3-year responders in subsequent supervised ML analyses.

PC1 weights revealed the main feature groups selected by the exhaustive feature selection and contributing to the correlation with the duration of surgical response. Of these, 12 were pain-related features, such as frequency of attacks (five features), degree of pre-surgical medication relief and character of pain episodes (the presence of spontaneous episodes was a negative predictor, while the presence of shock-like episodes was positive). Along with pre-surgical pain characteristics, individual medical history (history of hypothyroidism, type 2 diabetes, cancer) and patient sex were informative for the estimation and prognosis of surgical outcome (Fig. 2B).

### Brain imaging predictors of surgical outcome

**Figure 3:**
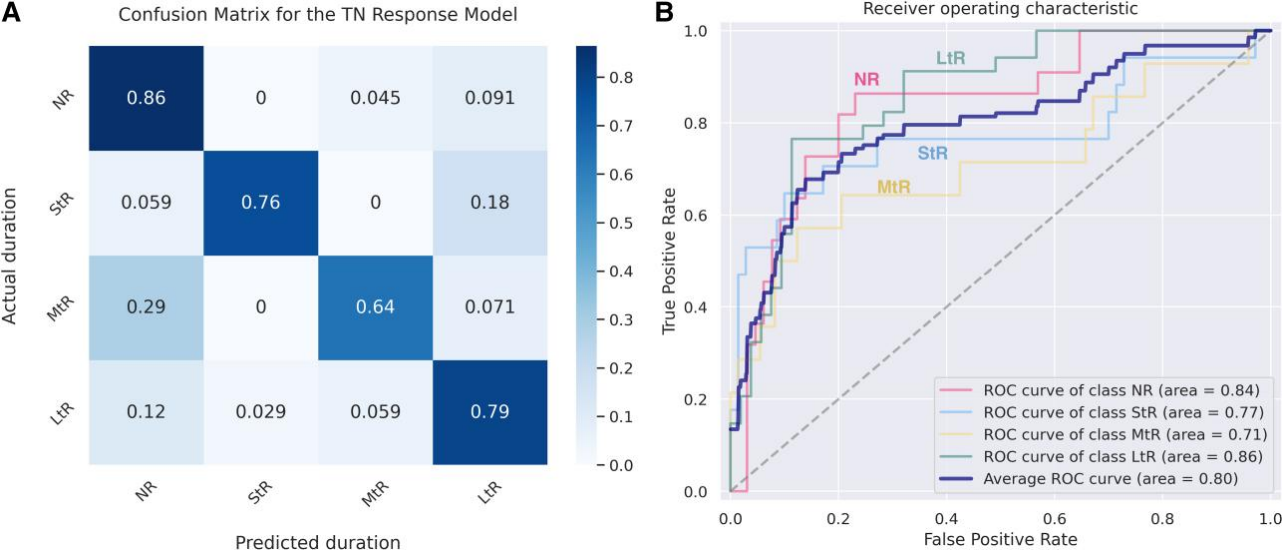
**SVM one-versus-rest (OvR) classifier performance.** Duration of response can be identified using GM metrics only cross-validation (CV) accuracy of 78% (*n* = 87). (**A**) Normalized confusion matrix (by true label) demonstrates the proportion of data classified correctly for each class. (**B**) Area under the receiver operating characteristics curve (AUC) for each class estimator and OvR classifier. AUC microaverage was derived from 87 predictions across 10 folds of CV and represents probability value of each category of surgical outcome. AUC, area under the receiver operating characteristics curve; GM, grey matter; OvR, one-versus-rest classifier; SVM, support vector machine; TN, trigeminal neuralgia.

A multiclass SVM classification model was trained on regional GM metrics from pre-surgical T1-weighted data. The model demonstrated a high recall for non-responders, short-term and long-term responders (0.86, 0.76 and 0.79). These values are higher than recall by chance (0.25 for the four-class classification). On average multiclass model achieved CV training accuracy of 95% and testing accuracy of 78% in predicting the duration of surgical response. The area under the cross-validated receiver operating characteristic curve for the multiclass one-versus-rest classifier was 0.8, with highest AUC for non-responders and long-term responders (0.84 and 0.86, respectively) (Fig. 3).

**Figure 4:**
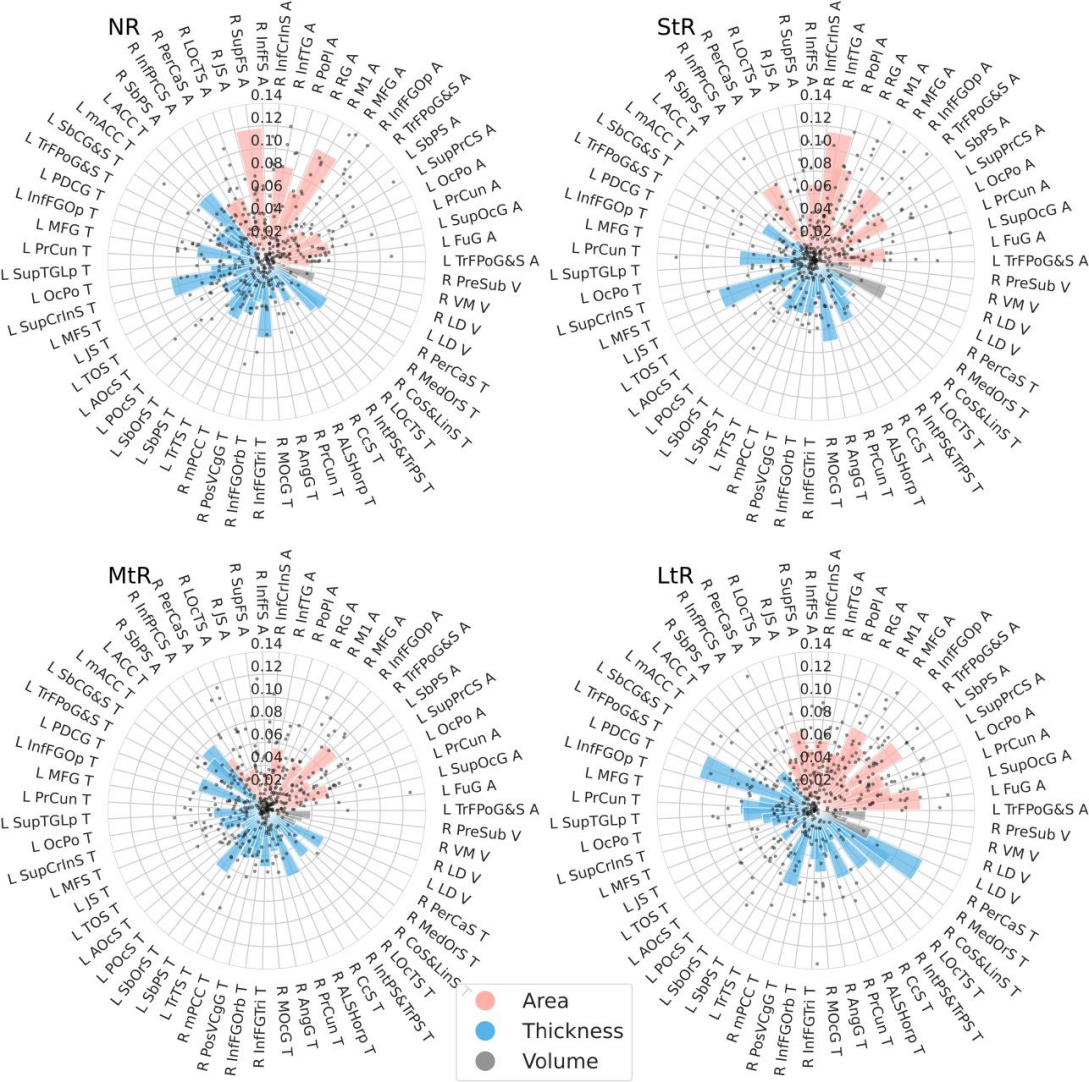
**One-versus-rest classifier feature importance derived from support vector machine (SVM) classification (*n* = 87 patients).** Each data point represents the feature importance derived from one classifier trained during 10-fold cross-validation (maximum of 10 data points per feature). A total of 59 cortical metrics from 53 brain regions are displayed. Feature importance values are unitless and indicate the relative contribution of each feature to the decision boundary for classification into non-responders (NR), short-term responders (StR), mid-term responders (MtR) and long-term responders (LtR). Cortical area, cortical volume, and cortical thickness importances are displayed. Feature importance values were SVM-derived; no statistical analyses were applied. NR, non-responders; StR, short-term (<1 year) responders; MtR, mid-term (1–3 year) responders; LtR, long-term (>3) year responders; L, left hemisphere; R, right hemisphere; A, surface area; T, thickness; V, volume. Abbreviations for brain regions are listed in the Supplementary Table 3.

A total of 368 GM metrics were identified during all 10 rounds of feature selection. Of these, 59 metrics from 53 brain regions, representing regional cortical thickness, cortical surface area and volume for the thalamic nuclei were consistently (six or more times) found important for predicting the duration of pain relief following surgery (Fig. 4). Each category estimator has a unique set of feature weights, allowing to distinguish these subjects from the rest of the study population. For NRs, the primary motor cortex (M1), right temporal cortex, right superior frontal sulcus (SupFS) surface area, left insula and anterior cingulate cortex (ACC) thickness measures were highlighted (Fig. 4). The best predictors for the StRs were surface area of the right inferior temporal gyrus (InfTG) and frontal cortex thickness. MtRs were identified mainly based on ACC and frontal pole. LtRs were distinguished on posterior cingulate regions (PDCG) thickness changes, pericallosal cortex thickness (Supplementary Fig. 1).

**Figure 5:**
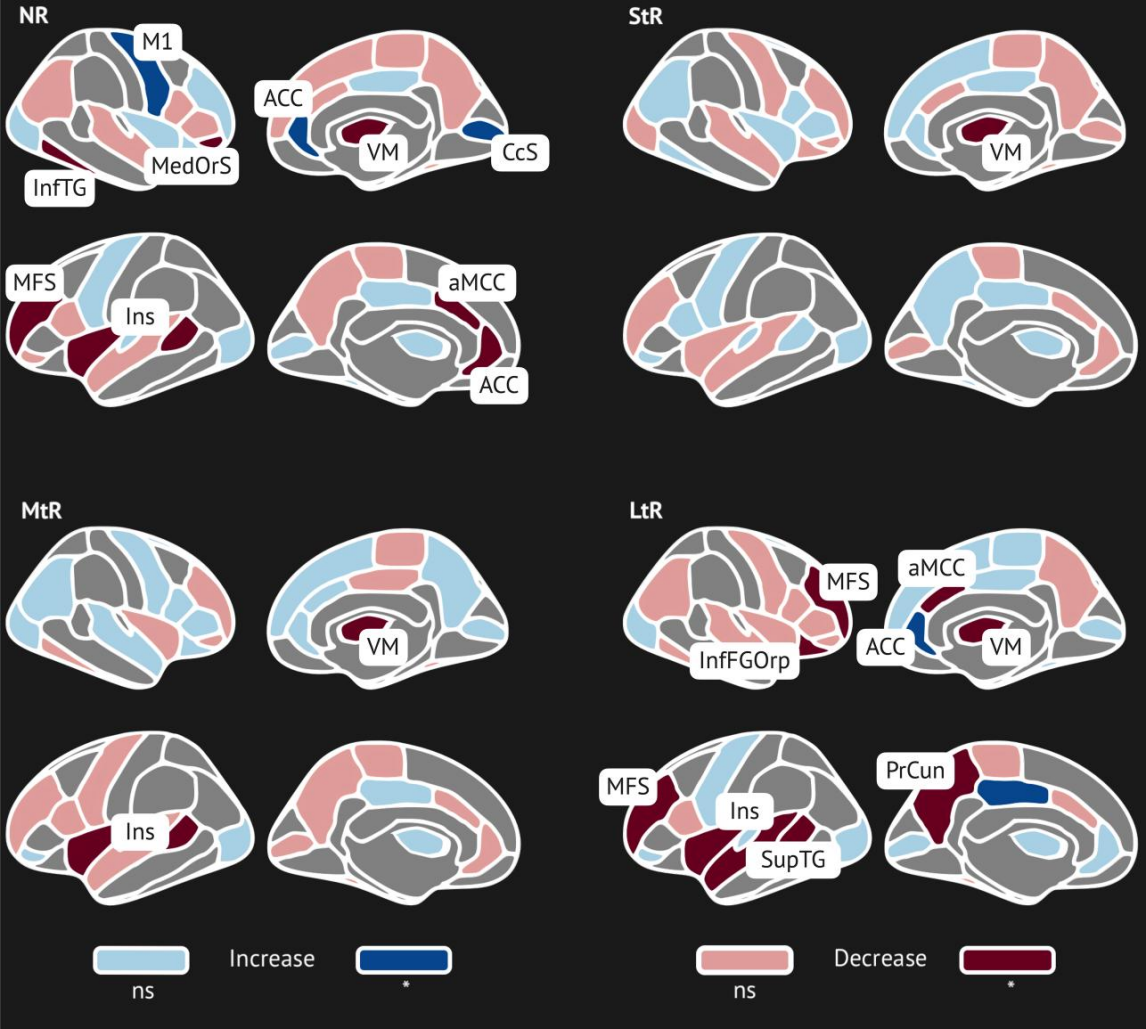
***Post hoc* univariate statistical comparison between TN and HC imaging data within ML-derived predictive regions.** Model pointed to brain structures that were affected in different categories of TN. Regions that show statistically significant differences between TN (*n* = 87) and HC (*n* = 87) groups (Mann–Whitney U-test was used, *P*-corrected < 0.05) are highlighted. ACC, anterior cingulate cortex; aMCC, anterior mid-cingulate cortex; CcS, calcarine sulcus; HC, healthy control; Ins, insula; InfFGOp, inferior frontal gyrus; InfTG, inferior temporal gyrus; M1, primary motor cortex; MedOrS, medial orbital sulcus (olfactory cortex); MFS, middle frontal sulcus; ns, non-significant; PDCG, posterior dorsal cingulate gyrus; PrCun, precuneus gyrus; SupTG, superior temporal gyrus; TN, trigeminal neuralgia; VM, ventromedial nucleus of thalamus.

*Post hoc* comparison between groups revealed that frontal, motor, cingulate cortex and visual cortex regions show a statistically significant difference (Supplementary Fig. 1). Univariate analysis of different TN categories and matched HCs showed that NRs had significantly reduced surface area in the left insula, left middle portion of anterior cingulate cortex (mACC) and left middle frontal cortex, as well as reduced ventromedial thalamus volume (VM) (corrected *P* < 0.05). Interestingly, a relative increase in M1 surface area, rostral ACC and occipital cortex thickness had been observed in NRs compared to HCs (corrected *P* < 0.05) (Fig. 5). StRs showed significant volume reduction in the VM. MtRs had significantly reduced left insula surface area and VM volume. LtRs also had significantly reduced left insula and frontal cortex thickness and an increase in dorsal cingulate cortex thickness (*P* < 0.05). Overall, we observe more statistically significant predictors in non-responders and long-term responders, which is also aligned with better performance of the multiclass classifier in identifying these categories.

## Discussion

The ICHD defines TN under category 13.1.1, which is further divided into subclasses based on the presence or absence of neurovascular compression (NVC) or secondary cause (13.1.1.2 Secondary TN).^1^ Patients with NVC are classified as having classical TN (13.1.1.1), whereas those without NVC fall under the idiopathic TN subclass (13.1.1.3). Both classical and idiopathic TN can present with episodic paroxysms of pain or a combination of paroxysmal and constant pain. Importantly, both groups of patients are usually seeking neurosurgical treatment.^4^ MVD is often offered to patients with classical TN, while GKRS and other ablative procedures may be considered for both classical and idiopathic TN, particularly in cases where NVC is absent or surgical risks are higher.^4,6,25^

The inherently subjective nature of chronic pain and the methods used to measure it presents challenges in deriving universally applicable measurements. This can affect the effectiveness and consistency of medical interventions and further highlights the need for novel methods to assess chronic pain. We present an ML-driven approach to stratify the intrinsic categories of TN using both unsupervised and supervised ML techniques. Our definition of categories is based on the duration of surgical response, a biologically relevant variable. This study builds on previous research that has compared responders and non-responders to the GK surgery in TN, as well as analysed pre-surgical brain imaging data from TN patients and HCs to differentiate TN from other forms of facial pain.^11-13^ In this work, we characterized not only differentiation between responders and non-responders but assessed the long-term outcome of surgical treatment of TN, providing means to forecast the duration of pain relief following surgery. Our analysis uncovered the influence of individual clinical data including medical history, sex, age and pain characteristics on surgical outcome and revealed clinical and imaging measures that can be used for the accurate prognosis for TN patients.

### Clinical prognosticators of duration of surgical response

We identified a set of clinical prognosticators of successful surgical intervention in TN. The identification of predictors of pain relief following surgery is crucial for improving patient outcomes and informing clinical decision-making. Similar to our results, previous studies uncovered that patients with typical TN pain presentation had a higher likelihood of experiencing pain freedom following surgery compared to patients with pain, characterized by a constant nature and spontaneous attacks.^26,27^ In addition, TN patients who had lower pre-operative pain frequency had better pain control after MVD than those who had higher pre-operative pain frequency.^28^ Our findings reinforce the need for a focus on attack frequency as important clinical parameter.

Although direct investigations on the relationship between medication relief and surgical outcome are yet to be conducted, previous studies demonstrated that patients with TN who experience poor relief from pharmacological therapy are more likely to have a longer disease duration and constant and/or spontaneous TN pain characteristics.^29^ Since constant pain has been widely linked to failed surgical outcomes,^26,27^ it is important for clinicians to consider the degree of pre-operative medication-induced pain relief. The identification of patients who are likely to benefit from surgery may help expedite the patient's journey from the initial referral to the diagnosis and treatment and help achieve complete pain relief following surgery.^5,30,31^

Along with pain characteristics, we found the important influence of individual medical history on pain relief following surgery in patients with TN. Consistent with our findings, a higher prevalence of diabetes mellitus in the TN group compared to the control group had been demonstrated previously.^32^ This suggests that painful neuropathy may exacerbate TN pathophysiology and negatively impact surgical response. The incidence of depression history was not frequent and did not have the high PC weight (Fig. 2B, Supplementary Table 2). However, its presence indicates that it may affect the course of TN, which is aligned with potential link between TN and post-traumatic stress disorder.^33,34^ Thyroid-related comorbidities were positive predictors of successful surgery in TN patients. It is important to state that there were no patients with thyroid-related comorbidities in the non-responder group (Supplementary Table 2). These results corroborate previously reported enhanced medical care for those with a higher comorbidity burden.^35^

In our study, the female sex was a positive predictor for a successful surgical response—female patients had a higher proportion of responders comping to males. However, this finding could be driven by the imbalanced sex ratio in our study cohort (Table 1). Previous studies on sex differences in TN have presented contrasting results, showing worse pain relief and a higher rate of recurrence in females.^6,25^ Similar to our results, younger age was shown to be a negative predictor of successful surgical outcomes.^25^

**Figure 6:**
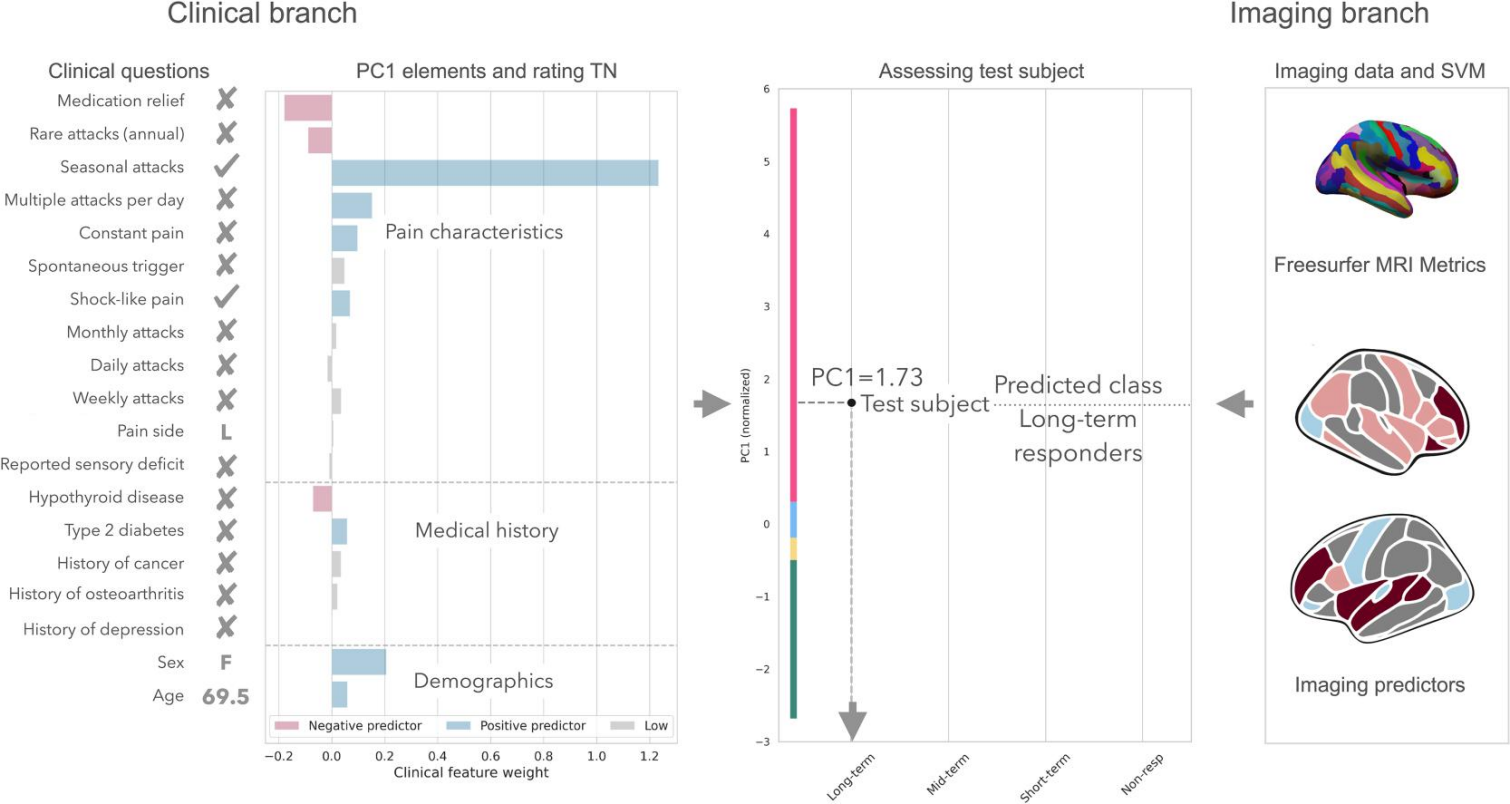
**An example of held-out subject categorization using novel TN assessment framework.** Pre-surgical clinical data and neuroimaging are used to estimate the category of surgical outcome. PCA, principal component analysis; PC, principal component; SVM, support vector machine.

In sum, we demonstrate which clinical features may serve as prognosticators of surgical intervention outcomes. Transformed clinical data can be used for novel ML approach to assess pain and screen patients. The example of how the correlation between patients’ PC1 scores with surgical outcomes can be leveraged for optimal treatment planning is shown in Fig. 6. This is critical as delay in diagnosis and referral is often reported and could result in worse surgical outcomes in TN patients.^5,36,37^

### Brain grey matter signatures of surgical outcome

The multiclass classification model performed with high accuracy and identified a set of unique neuroimaging grey matter metrics that are predictive for the duration of surgical response in TN (Fig. 5, Supplementary Fig. 1). Key regions include thalamic nuclei, insula, cingulate cortex, hippocampus and frontal and temporal regions.

Identification of thalamic nuclei, known for their roles in sensory and affective pain components,^38^ as important predictor aligns with previous investigations of thalamic atrophy in TN patients that also was associated with higher pre-operative pain frequency.^39^ Structural and functional thalamic alterations were also identified in TN patients compared to HCs.^40^

Cingulate cortex and insula alterations were indicative for distinguishing NRs and LtRs. The insula's significance in pain processing is evident in our study; its cortical thickness stood out for distinguishing NRs. These findings were consistent with previous studies that showed similar structural changes in both insular cortex and cingulate cortex in TN.^41-43^ Similar pattern can be observed in temporal cortex regions.^12,13^ Moreover, prior research noted the volumetric changes in temporal cortex being correlated with duration of pain.^44,45^

The prefrontal and orbitofrontal cortex areas are highly involved in pain processing and biopsychosocial pain management due to their connections to other areas of the neocortex, hippocampus, periaqueductal grey, thalamus, amygdala and basal nuclei.^46,47^ Our findings reinforce the TN-associated alterations in the frontal cortex area and frontal pole revealed in our analysis, mirroring previous studies pinpointing predictive nature of these regions for the outcome of surgery.^12^

Contrary to common expectation of GM loss, we observed increased surface area in M1 (Fig. 5). Motor and pre-motor cortices were reported to be an important site for the neuromodulation, particularly, chronic pain treatment.^48,49^ In addition, motor abnormalities and pain are highly associated, and this typically is aligned with motor regions being affected in chronic pain population.^50^

Altogether, these findings pinpoint several imaging-based biomarkers of specific surgical outcomes in TN. The relationship between brain structural abnormalities and TN is not clearly defined as observed changes may be both caused by TN and predispose individuals to it.^51^ Similarly to other chronic pain conditions, central sensitization and cortical reorganization were proposed as the underlying mechanisms of TN pathogenesis.^52^ The affected structures mainly include ‘pain matrix’ regions—a system of brain networks heavily involved in the multidimensional processing of pain.^53^

### Towards a definition of stages of trigeminal neuralgia pain

While classical TN is highlighted by a fairly homogenous collection of clinical symptoms, further analysis reveals that indeed patients may report a range of symptoms, which may include paroxysmal and constant pain, reduction in the efficacy of medication relief^7,54^ and at times the development of sensory abnormalities. As reported by Burchiel and Slavin,^7^ there appears to be a temporal pattern to these symptoms, such that early on in the diagnosis, patients are far more likely to have occasional pain and good response to medications. With time, the response to medications decreases, and pain attacks become more frequent.

It is plausible that in fact TN consists of distinct stages, each associated with specific expression of pain, such as frequency of attacks, character of TN pain, degree of medication relief and surgical outcome (Fig. 2). We propose that these stages in TN would follow each other sequentially, even though the timeline of movement through these stages may vary in different individuals, as suggested in previous longitudinal studies focused on evaluating progressive nature of TN.^55,56^ The temporal evolution of TN pain may be influenced by other factors that include demographics, age, as well as medical comorbidities including diabetes.^2,54^ While this analysis does not focus directly on the natural history of TN, important clinical characteristics uncovered in this study have been previously shown as signatures of TN progression.^7,55^ For instance, the decrease in medication efficacy, the increase in the frequency of attacks, changes in pain character and the development of sensory abnormalities were reported in the longitudinal observations of TN patients.^7,55,57^ The evidence of within-subjects progression of TN pain together with our results showing clinical categories of TN surgical outcome opens the pathway for the defining TN stages with the connection to the treatment outcome. Importantly, our present work serves as a holistic analysis that takes into account all of these parameters to draw predictors of response to surgical treatment.

### Study limitations

This study has limitations that should be considered when interpreting the results. Firstly, our retrospective study allows us to precisely characterize the outcome of surgical procedures but may be subject to bias due to the nature of clinical data. The set of clinical features of interest was defined based on the retrospective data, and it is possible that other important clinical tests that could have been included in the analysis were not available. Additionally, we characterized the degree of medication relief; however, we cannot completely rule out the medication effects on brain structure.

Our study was conducted using single-centre data from 102 subjects. While the sample size requirements for the imaging studies are still an ongoing discussion,^58,59^ recent studies provide compelling evidence that moderate sample sizes (*n* > 75) can still provide reliable and accurate insights.^59^ Ten-fold CV evaluation demonstrated reasonable estimate for performance of ML models on data collected from our institution. This work sets a foundation for the development of large multimodal cross-centre cohorts of that can be used to confirm the generalizability of the trained models.

We acknowledge that the definition of initial surgical response as 75% reduction in pain intensity and BNI pain scale reduction down to I–III can be considered as arbitrary. We set a strict criterion for the definition of pain relief, to emphasize that patients with TN generally have very good pain relief profile. This allowed us to minimize data fragmentation and facilitated a more robust and generalizable analysis. We also acknowledge that there may be differences in the effects of GK and MVD surgeries, including but not limited to the duration of procedure effects and possible placebo effects. Given difference in mechanisms and different contraindications for these procedures, we cannot fully account for potential biases in comparing the two treatment methods. Noting this point however, we accounted for the difference in the onset of pain relief for MVD and GK procedures. This approach allows us to assess response in more diverse population, therefore providing insights from a wider cohort of TN patients.

This study separated clinical and imaging data analyses as the complexity of interactions between the features may not be modelled accurately together, resulting in a bias towards a specific data modality. These independent but complementary findings may not be directly comparable but can inform the development of clinical and imaging-based TN assessment methods.

## Summary

This study confirms the stratification of different categories of surgical response in patients with TN, using clinical data, neuroimaging and ML. Our findings present a promising avenue for extrapolation of TN findings to the broader chronic pain population, and these insights have a potential for forecasting clinical outcomes across various pain conditions. The increasing body of work on the neuroimaging in chronic pain, combined with ML methods that include clinical and imaging parameters, provides a foundation for a better understanding of the nature of chronic pain, and paves the way for developing innovative methods for assessing pain and surgical outcome in these patients.

## Supplementary Material

fcaf178_Supplementary_Data

## Data Availability

MR data from trigeminal pain subjects can be shared upon reasonable request. Code for the analysis is available online (https://github.com/latypovt/TN_Categories.git). The CamCAN imaging data are publicly available (https://camcan-archive.mrc-cbu.cam.ac.uk/dataaccess/index.php).
