# Supplementary material for: Stratification of surgical outcomes in trigeminal neuralgia using multimodal data
Source: Brain Commun. 2025 Jun 17;7(3):fcaf178. doi: 10.1093/braincomms/fcaf178 (PMC12199914; doi:10.1093/braincomms/fcaf178)
Supplement: fcaf178_Supplementary_Data [file fcaf178_supplementary_data.docx]

Supplementary material:

**P1.Unsupervised Machine Learning for feature extraction on clinical data**

For the purposes of the machine learning (ML)-driven analysis, categorical clinical data was converted into numeric ordinal data, dichotomous data, and one-hot encoded. Z-score normalization was applied to all features prior to the transformation. We used Principal Component Analysis (PCA) to transform clinical data into continuous variables (principal component, PC) using linear transformation ^1,2^. Each principal component (PC) represents the entire dataset with an emphasis on variables that produce the most variation^1^. Feature extraction was conducted through an exhaustive feature selection that optimized the correlation of PCs with the duration of surgical response. This approach computes all possible combinations of the 36 clinical features and identifies the set of features with the highest Spearman’s rank correlation of PC1 with the duration of surgical response. We chose PC1 as this is the component that explains the maximum data variance in one dimension.

**P2. Supervised ML learning on MRI data**

We constructed a supervised ML model which follows the One-vs-Rest multiclass classification strategy to distinguish different classes of surgical response. Linear support vector machine classifiers were used as base estimators for the One-vs-Rest model. Model parameters: linear kernel, gamma = ‘scale’, C=1, tol=0.001, class weight = balanced. C, gamma, tol parameters were default. ML algorithms and data preprocessing steps were implemented using Scikit-learn (v.1.22; <http://scikit-learn.org>). Similar to our previous work (Hung et. al, 2021), we performed a backward sequential feature selection using ‘mlxtend’ framework (<http://rasbt.github.io/mlxtend/>). After performing feature selection, we used 10-fold cross-validation for model performance evaluation^3^.

**P3. Statistical analyses**

To identify the directionality of regional changes as significant neuroimaging predictors of surgical response, we compared regional GM metrics between TN subjects and age- and sex-matched healthy controls (HC) from Cambridge Center for Ageing Neuroscience dataset (n=87)^4^. Student’s independent T-Test was used to compare the regional metrics between TN and HC data. In addition, we tested imaging predictors between different TN categories using Mann-Whitney non-parametric test. The univariate comparison between pain subjects followed the One-vs-Rest schema. Two-proportion z-test was used to compare proportions of surgical outcome between GK and MVD. P-values for all univariate statistical tests and Spearman correlation (of PCs) were corrected for multiple tests using a false discovery rate procedure.

**Supplementary table 1.** Clinical variables and the usage of features

| No. | Variable | Description | Values | Usage and the number of features |
| --- | --- | --- | --- | --- |
|  | **Pain characteristics** | | | |
| 1 | Pain laterality | Side of TN pain. | Right, left | Categorical (1 feature) |
| 2 | Duration of TN | Years since the onset of TN symptoms | Integer | Continuous, z-score normalized (1 feature) |
| 3 | Description of pain | Self-reported description of pain at pre-surgical follow-up. | Shocking, burning, dull | One-hot-encoded (3 features) |
| 4 | Triggers | Self-reported triggers of pain; ‘typically’ triggered pain is associated with mechanical stimulation; ‘atypically’ triggered pain is spontaneous | Typical, spontaneous pain episodes (no triggers) | Categorical (1 feature) |
| 5 | Attack frequency | Frequency of TN pain attacks experienced at pre-surgical follow-up. | Rare (annual), seasonal, monthly, weekly, daily (1), multiple per day (2 and more), constant | One-hot-encoded categories (6 features) |
| 6 | CN V division(s) affected | CN V branches affected. | V1, V2, V3 | One-hot-encoded categories (3 features) |
| 7 | CN V deficit(s) | Self reported CN V sensory deficits (numbness, decreased sensation) | Yes, no | Categorical (1 feature) |
| 8 | Pain medication effect | Degree of relief from pain medication patient at time of pre-surgical follow-up. | Relief, partial relief, non-responder | One-hot-encoded categories (3 features) |
|  | **Medical history** | | | |
| 9 | Neurosurgical history | History of neurosurgical intervention | Yes, no | Categorical (1 feature) |
| 10 | GK history | History of previously done GKRS | Yes, no | Categorical (1 feature) |
| 11 | MVD history | History of previously done MVD | Yes, no | Categorical (1 feature) |
| 12 | Arthritis | Documented history of disease | Yes, no | Categorical (1 feature) |
| 13 | Hypothyroid comorbidities | Documented history of disease | Yes, no | Categorical (1 feature) |
| 14 | Urogenital comorbidities | Documented history of disease | Yes, no | Categorical (1 feature) |
| 15 | Autoimmune comorbidities | Documented history of disease | Yes, no | Categorical (1 feature) |
| 16 | Cardiological comorbidities | Documented history of disease | Yes, no | Categorical (1 feature) |
| 17 | Respiratory comorbidities | Documented history of disease | Yes, no | Categorical (1 feature) |
| 18 | Gastrointestinal comorbidities | Documented history of disease | Yes, no | Categorical (1 feature) |
| 19 | Cancer(s) | Documented history of disease | Yes, no | Categorical (1 feature) |
| 20 | Depression/anxiety | Documented history of disease | Yes, no | Categorical (1 feature) |
| 21 | Hypertension | Documented history of disease | Yes, no | Categorical (1 feature) |
| 22 | Dyslipidemia | Documented history of disease | Yes, no | Categorical (1 feature) |
| 23 | Diabetes | Documented history of disease | Yes, no | Categorical (1 feature) |
|  | **Demographic** | | | |
| 24 | Age | Age of patient at time of pre-surgical follow-up. | Integer value | Continuous, z-score normalized (1 feature) |
| 25 | Sex | Sex of patient (as indicated in electronic patient records). | Male, female | Categorical (1 feature) |
|  |  |  | **Total** | **36** |

**Supplementary Table 2.** Group information on each clinical parameter.

| Feature | Non responders | Short-term | Mid-term | Long-term (3-5 y) | Long-term ( >5y) |
| --- | --- | --- | --- | --- | --- |
| N | 33 | 21 | 17 | 11 | 20 |
| Cancer | 4 | 1 | 2 | 0 | 1 |
| Constant pain | 9 | 2 | 3 | 2 | 3 |
| Daily attacks | 7 | 1 | 4 | 5 | 6 |
| Diabetes | 4 | 3 | 3 | 0 | 0 |
| Electric pain | 27 | 16 | 16 | 10 | 17 |
| Medication relief | 1 | 1 | 1 | 2 | 4 |
| Monthly attacks | 2 | 1 | 2 | 1 | 0 |
| Osteoarthritis | 5 | 3 | 2 | 0 | 2 |
| Mutliple attacks | 11 | 13 | 4 | 1 | 4 |
| Rare attacks | 0 | 1 | 1 | 0 | 2 |
| Pain side | 8 | 9 | 11 | 3 | 6 |
| Depression | 1 | 1 | 0 | 0 | 0 |
| Seasonal attacks | 1 | 2 | 0 | 2 | 4 |
| Sex | 14 | 12 | 13 | 6 | 14 |
| Thyroid | 0 | 2 | 1 | 2 | 4 |
| Trigeminal deficit | 11 | 7 | 5 | 2 | 2 |
| Triggers | 5 | 2 | 0 | 0 | 0 |
| Weekly attacks | 3 | 1 | 3 | 0 | 1 |

**Supplementary Table 3.** Abbreviation and corresponding brain region name.

| **Region Abbreviation** | **Region Name** |
| --- | --- |
| ACC | Anterior cingulate cortex |
| ACrInS | Anterior circular insular sulcus |
| ALSHorp | Anterior horizontal ramus of lateral fissure |
| aMCC | Mid-anterior cingulate cortex |
| AngG | Angular gyrus |
| AOcS | Anterior occipital sulcus |
| CcS | Calcarine sulcus |
| CoS&LinS | Collateral and lingual sulcus |
| FuG | Fusiform gyrus |
| InfCrInS | Inferior circular insular sulcus |
| InfFGOp | Inferior frontal gyrus, opercular part |
| InfFGOrb | Inferior frontal gyrus, orbital part |
| InfFGTri | Inferior frontal gyrus, triangular part |
| InfFS | Inferior frontal sulcus |
| InfPrCS | Inferior precentral sulcus |
| InfTG | Inferior temporal gyrus |
| IntPS&TrPS | Intraparietal and transverse parietal sulci |
| JS | Jensen sulcus |
| LD | Lateral dorsal nucleus (thalamus) |
| LOcTS | Lateral occipito-temporal sulcus |
| M1 | Primary motor cortex (precentral gyrus) |
| MedOrS | Medial orbital sulcus |
| MFG | Middle frontal gyrus |
| MFS | Middle frontal sulcus |
| MOcG | Middle occipital gyrus |
| mPCC | Mid-posterior cingulate cortex |
| OcPo | Occipital pole |
| PDCG | Posterior dorsal cingulate gyrus |
| PerCaS | Pericallosal sulcus |
| POcS | Parieto-occipital sulcus |
| PoPl | Planum polare |
| PosVCgG | Posterior ventral cingulate gyrus |
| PrCun | Precuneus |
| PreSub | Presubiculum |
| RG | Rectus gyrus |
| SbCG&S | Subcentral gyrus and sulcus |
| SbOrS | Suborbital sulcus |
| SbPS | Subparietal sulcus |
| SupCrInS | Superior circular insular sulcus |
| SupFS | Superior frontal sulcus |
| SupOcG | Superior occipital gyrus |
| SupPrCS | Superior precentral sulcus |
| SupTGLp | Superior temporal gyrus, lateral part |
| TOS | Transverse occipital sulcus |
| TrFPoG&S | Transverse frontopolar gyri and sulci |
| TrTS | Transverse temporal sulcus |
| VM | Ventral medial nucleus (thalamus) |

**
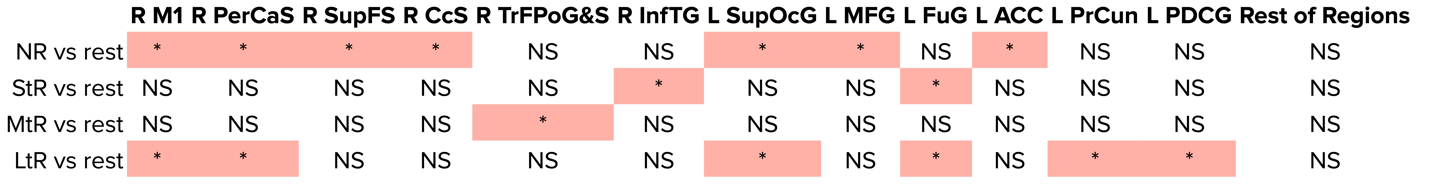
**

**Supplementary Figure 1.** Univariate statistics comparing important predictors of surgical outcome categories – One-vs-Rest comparison. Mann-Whitney Test was used, p-values are corrected for multiple comparisons using Benjamini-Hochberg false discovery rate. * - p<0.05, NS – p>0.05 (n=87, NR: 22, StR:17, MtR:14, LtR:34). NR - Non-responders, StR – short-term (<1 year) responders, MtR – mid-term (1-3 year) responders, LtR – long-term (>3) year responders.

**Reference**

1. Ringnér, M. What is principal component analysis? *Nat. Biotechnol.* **26**, 303–304 (2008).

2. Zhang, Z. & Castelló, A. Principal components analysis in clinical studies. *Ann. Transl. Med.* **5**, 351 (2017).

3. Varoquaux, G. *et al.* Assessing and tuning brain decoders: Cross-validation, caveats, and guidelines. *Individ. Subj. Predict.* **145**, 166–179 (2017).

4. Taylor, J. R. *et al.* The Cambridge Centre for Ageing and Neuroscience (Cam-CAN) data repository: Structural and functional MRI, MEG, and cognitive data from a cross-sectional adult lifespan sample. *NeuroImage* **144**, 262–269 (2017).
